# Supplementary material for: Polyketide synthase gene clusters in the ericoid mycorrhizal fungus Oidiodendron maius: genome-wide prediction, expression profiling, and functional characterization of knockout mutants
Source: Front Plant Sci. 2026 Sep 16;17:1924430. doi: 10.3389/fpls.2026.1924430 (PMC13623903; doi:10.3389/fpls.2026.1924430)
Supplement: Supplementary file 1 [file SupplementaryFile1.zip › Supplementary Materials/Supplementary Folder 1.zip!/Oidma1_AssemblyScaffolds_Repeatmasked/knownclusterblast/cluster4/jgi.p_Oidma1_36690_mibig_hits.html]

| MIBiG Protein | Description | MIBiG Cluster | MiBiG Product | % ID | % Coverage | BLAST Score | E-value |
| --- | --- | --- | --- | --- | --- | --- | --- |
| EAQ86388.1 | hypothetical\_protein | BGC0001405 | Chaetoviridin\_/\_chaetomugilin\_biosynthetic\_gene\_cluster | 61 | 92.1328671329 | 638 | 6.1e-183 |
| BAE56588.1 |  | BGC0001123 | Aspirochlorine\_biosynthetic\_gene\_cluster | 59 | 91.6083916084 | 636 | 2.3e-182 |
| AGO59046.1 | PtaM | BGC0000121 | Pestheic\_acid\_biosynthetic\_gene\_cluster | 53 | 96.3286713287 | 581 | 8.8e-166 |
| ACD39771.1 | non-heme\_halogenase | BGC0000134 | Radicicol\_biosynthetic\_gene\_cluster | 52 | 93.3566433566 | 535 | 5.5e-152 |
| ADI24948.1 | GsfI | BGC0000070 | Griseofulvin\_biosynthetic\_gene\_cluster | 51 | 93.1818181818 | 532 | 6.1e-151 |
| AEM76785.1 | putative\_halogenase | BGC0001401 | Melleolides\_biosynthetic\_gene\_cluster | 48 | 93.006993007 | 489 | 5.9e-138 |
| ALT31850.1 | ArmH3 | BGC0001401\_c3 | Melleolides\_biosynthetic\_gene\_cluster | 48 | 92.6573426573 | 475 | 1.2e-133 |
| AEM76786.1 | putative\_halogenase | BGC0001401\_c2 | Melleolides\_biosynthetic\_gene\_cluster | 45 | 94.2307692308 | 464 | 1.6e-130 |
| ALT31851.1 | ArmH4 | BGC0001401\_c4 | Melleolides\_biosynthetic\_gene\_cluster | 48 | 94.0559440559 | 461 | 1.7e-129 |
| ALT31852.1 | ArmH5 | BGC0001401\_c5 | Melleolides\_biosynthetic\_gene\_cluster | 46 | 92.4825174825 | 449 | 6.8e-126 |
| ADE22292.1 | putative\_non-heme\_halogenase | BGC0000279 | Xantholipin\_biosynthetic\_gene\_cluster | 37 | 73.2517482517 | 266 | 6.3e-71 |
| AMK51270.1 | Hex13 | BGC0001376 | Hexaricin\_biosynthetic\_gene\_cluster | 35 | 75.8741258741 | 260 | 4.5e-69 |
| ABM21576.1 | CrpH | BGC0000975 | Cryptophycin\_biosynthetic\_gene\_cluster | 36 | 67.8321678322 | 259 | 7.6e-69 |
| CAM34371.1 | putative\_non-heme\_halogenase | BGC0000242 | Lysolipin\_biosynthetic\_gene\_cluster | 37 | 73.2517482517 | 251 | 2.1e-66 |
| CAJ46693.1 | tryptophan\_halogenase | BGC0000969 | Chondramide\_biosynthetic\_gene\_cluster | 37 | 69.2307692308 | 245 | 1.5e-64 |
| ACU71639.1 | tryptophan\_halogenase | BGC0001154 | Cacibiocin\_biosynthetic\_gene\_cluster | 32 | 66.6083916084 | 208 | 2.6e-53 |
| WP\_052390547.1 | halogenase | BGC0001308 | Simocyclinone\_D9\_/\_simocyclinone\_D10\_/\_simocyclinone\_D11 | 30 | 74.4755244755 | 207 | 4.5e-53 |
| AEU17894.1 | putative\_halogenase | BGC0001072 | Simocyclinone\_biosynthetic\_gene\_cluster | 30 | 74.4755244755 | 206 | 1e-52 |
| ADU85999.1 | putative\_tryptophan\_halogenase | BGC0000165 | Tiacumicin\_B\_biosynthetic\_gene\_cluster | 29 | 66.7832167832 | 188 | 2.8e-47 |
| AAN65237.1 | putative\_halogenase | BGC0000832 | Clorobiocin\_biosynthetic\_gene\_cluster | 33 | 61.1888111888 | 176 | 8.5e-44 |
| ctg1\_orf12 |  | BGC0001366 | BE-24566B\_/\_zunyimycin\_A\_biosynthetic\_gene\_cluster | 32 | 67.1328671329 | 176 | 1.1e-43 |
| AAK81830.1 | non-heme\_halogenase | BGC0000326 | Complestatin\_biosynthetic\_gene\_cluster | 31 | 69.9300699301 | 175 | 1.5e-43 |
| AEI58871.1 | halogenase | BGC0000455 | Vancomycin\_biosynthetic\_gene\_cluster | 30 | 68.7062937063 | 173 | 9.4e-43 |
| CAA76550.1 | halogenase | BGC0000311 | Balhimycin\_biosynthetic\_gene\_cluster | 30 | 68.7062937063 | 170 | 6.1e-42 |
| CAA11780.1 | PCZA361.26 | BGC0000322\_c2 | Chloroeremomycin\_biosynthetic\_gene\_cluster | 30 | 68.7062937063 | 168 | 2.3e-41 |
| AGC09515.1 | FAD\_dependent\_halogenase | BGC0001183 | Lobophorin\_biosynthetic\_gene\_cluster | 30 | 66.2587412587 | 166 | 1.2e-40 |
| AFV52210.1 | aryl-ACP\_halogenase | BGC0000081 | Kedarcidin\_biosynthetic\_gene\_cluster | 31 | 65.5594405594 | 163 | 7.5e-40 |
| AAM80532.1 | StaI | BGC0000290 | A47934\_biosynthetic\_gene\_cluster | 30 | 64.3356643357 | 161 | 2.2e-39 |
| AAL06656.1 | alkylhalidase | BGC0000965 | C-1027\_biosynthetic\_gene\_cluster | 29 | 66.958041958 | 161 | 2.8e-39 |
| CAE53362.1 | halogenase | BGC0000440 | Teicoplanin\_biosynthetic\_gene\_cluster | 29 | 68.5314685315 | 161 | 3.7e-39 |
| CAG15020.1 | halogenase | BGC0000441 | Teicoplanin\_biosynthetic\_gene\_cluster | 29 | 68.5314685315 | 161 | 3.7e-39 |
| AGS77325.1 | Halogenase | BGC0001178 | UK-68,597\_biosynthetic\_gene\_cluster | 25 | 87.0629370629 | 160 | 8.2e-39 |
| ALU98528.1 | tryptophan\_halogenase | BGC0001397 | Lidamycin\_biosynthetic\_gene\_cluster | 30 | 65.7342657343 | 159 | 1.4e-38 |
| CAD91205.1 | halogenase | BGC0000289 | A40926\_biosynthetic\_gene\_cluster | 29 | 64.3356643357 | 158 | 3.1e-38 |
| ADM46362.1 | halogenase | BGC0000106 | Naphthomycin\_biosynthetic\_gene\_cluster | 29 | 66.0839160839 | 156 | 9.1e-38 |
| ABD65950.1 | halogenase | BGC0000341 | Enduracidin\_biosynthetic\_gene\_cluster | 29 | 64.1608391608 | 155 | 1.6e-37 |
| ABY66006.1 | halogenase | BGC0001008 | Maduropeptin\_biosynthetic\_gene\_cluster | 30 | 66.7832167832 | 155 | 2e-37 |
| AFP87533.1 | FADH2-dependent\_halogenase | BGC0001159 | Marinopyrrole\_biosynthetic\_gene\_cluster | 32 | 63.6363636364 | 149 | 1.1e-35 |
| ALD82533.1 | halogenase | BGC0001212 | Nannocystin\_biosynthetic\_gene\_cluster | 29 | 67.1328671329 | 149 | 1.5e-35 |
| ABS50460.1 | NapH2 | BGC0001079 | Napyradiomycin\_biosynthetic\_gene\_cluster | 27 | 59.4405594406 | 146 | 7.2e-35 |
| ABS50488.1 | NapH2 | BGC0000652 | Napyradiomycin\_biosynthetic\_gene\_cluster | 27 | 59.4405594406 | 146 | 7.2e-35 |
| AAM54090.1 | halogenase | BGC0000020 | Ansamitocin\_biosynthetic\_gene\_cluster | 30 | 66.2587412587 | 145 | 1.6e-34 |
| AHA12077.1 | flavin-dependent\_halogenase | BGC0001172 | Chlorizidine\_A\_biosynthetic\_gene\_cluster | 29 | 64.5104895105 | 142 | 1.4e-33 |
| CAQ43074.1 | halogenase | BGC0000970 | Chondrochloren\_biosynthetic\_gene\_cluster | 29 | 68.5314685315 | 141 | 4e-33 |
| AGS77327.1 | Halogenase | BGC0001178 | UK-68,597\_biosynthetic\_gene\_cluster | 27 | 67.8321678322 | 140 | 6.8e-33 |
| AAM80530.1 | StaK | BGC0000290 | A47934\_biosynthetic\_gene\_cluster | 27 | 64.1608391608 | 133 | 6.3e-31 |
| AAQ90172.1 | putative\_halogenase | BGC0000128 | Pyoluteorin\_biosynthetic\_gene\_cluster | 29 | 67.6573426573 | 129 | 9.1e-30 |
| AAM70353.1 | CalO3 | BGC0000033 | Calicheamicin\_biosynthetic\_gene\_cluster | 29 | 62.4125874126 | 127 | 4.5e-29 |
| ESP90845.1 | Dehydrogenases\_(flavoprotein) | BGC0000891 | Bromophenols\_/\_bromopyrroles\_biosynthetic\_gene\_cluster | 28 | 62.5874125874 | 126 | 7.7e-29 |
| AHA34035.1 | Bmp2 | BGC0000890 | Bromophenols\_/\_bromopyrroles\_biosynthetic\_gene\_cluster | 28 | 61.3636363636 | 123 | 1.1e-27 |
| AAD24884.1 | putative\_halogenase | BGC0000127 | Pyoluteorin\_biosynthetic\_gene\_cluster | 26 | 64.6853146853 | 104 | 4.1e-22 |
| ABQ04143.1 | tryptophan\_halogenase | BGC0000838 | Flexirubin\_biosynthetic\_gene\_cluster | 25 | 60.8391608392 | 98 | 2.3e-20 |
